# Modulating TRAP-mediated transcription termination by AT during transcription of the leader region of the *Bacillus subtilis trp* operon

**DOI:** 10.1093/nar/gku211

**Published:** 2014-03-20

**Authors:** Shraddha Sharma, Paul Gollnick

**Affiliations:** Department of Biological Sciences, University at Buffalo, The State University of New York, Buffalo, NY 14260, USA

## Abstract

An 11-subunit protein called *trp* RNA binding Attenuation Protein (TRAP) controls attenuation of the tryptophan biosynthetic (*trpEDCFBA*) operon in *Bacillus subtilis*. Tryptophan-activated TRAP binds to 11 (G/U)AG repeats in the 5′ leader region of *trp* mRNAs, and downregulates expression of the operon by promoting transcription termination prior to the structural genes. Anti-TRAP (AT) is an antagonist that binds to tryptophan-activated TRAP and prevents TRAP from binding to RNA, thereby upregulating expression of the *trp* genes. AT forms trimers, and multiple trimers bind to a TRAP 11mer. It is not known how many trimers must bind to TRAP in order to interfere with RNA binding. Studies of isolated TRAP and AT showed that AT can prevent TRAP from binding to the *trp* leader RNA but cannot dissociate a pre-formed TRAP-RNA complex. Here, we show that AT can prevent TRAP-mediated termination of transcription by inducing dissociation of TRAP from the nascent RNA when it has bound to fewer than all 11 (G/U)AG repeats. The 5′-most region of the TRAP binding site in the nascent transcript is most susceptible to dissociation from TRAP. We also show that one AT trimer bound to TRAP 11mer reduces the affinity of TRAP for RNA and eliminates TRAP-mediated transcription termination *in vitro*.

## INTRODUCTION

Regulating gene expression by transcription attenuation is a common strategy employed by bacteria (1). In *Bacillus subtilis* the *trp* RNA binding Attenuation Protein (TRAP) regulates transcription of the *trpEDCFBA* operon through an attenuation mechanism in response to changes in the intracellular concentration of tryptophan (2). TRAP consists of 11 identical 75 amino acid subunits, each encoded by the *mtrB* gene, arranged in a symmetrical ring (3,4). When intracellular tryptophan levels are high, TRAP is activated to bind to 11(G/U)AG triplet repeats in the 5′ leader region of the *trp* mRNA. Each (G/U)AG repeat interacts with Glu36 and Lys 37 from one subunit, and Lys 56 and Arg 58 from the adjacent subunit of TRAP (4,5). The presence of TRAP bound to the nascent *trp* transcript induces transcription termination prior to the structural genes of the operon (6–9).

In addition to responding to changes in the levels of free tryptophan, expression of the *trp* operon is also controlled in response to the degree of aminoacylation of tRNA^Trp^ (10,11). This regulation is mediated by a protein antagonist of TRAP called Anti-TRAP (AT). AT is produced in response to the accumulation of uncharged tRNA^Trp^. Both transcription and translation of AT are induced by elevated levels of uncharged tRNA^Trp^ (11,12). AT contains two cysteine-rich zinc-binding motifs similar to that of the Type I family of Hsp40 molecular chaperones (13–16). The four cysteine residues in AT coordinate a single Zn^2+^ atom, which is required for assembly and activity of AT (14,15). AT forms cone-shaped trimers with the N-terminal residues of each monomer at the apex of the trimer and the zinc-binding domains at the base (15).

AT binding prevents TRAP from binding to its target RNA (17), thereby increasing *trp* gene expression. Structural studies have shown that up to five AT trimers can simultaneously bind to a single tryptophan-activated TRAP 11mer, and six AT trimers bind an artificial TRAP 12mer (18). The top region of AT interacts with residues located in the RNA binding region of TRAP (19) with each AT trimer binding to two adjacent TRAP subunits within the ring (18).

Binding studies using purified AT, TRAP and *trp* leader RNA have shown that AT functions by directly competing with RNA for binding to TRAP (17,19). These studies indicate that AT cannot induce RNA to dissociate after it has bound to TRAP (17). However, these TRAP-RNA complexes may not accurately reflect the situation *in vivo* where TRAP must bind to the nascent *trp* transcript during transcription of the leader region in order to regulate transcription of the *trp* operon (2). Hence, we have examined whether AT can modulate TRAP-mediated control of attenuation during transcription of the *trp* leader region. To do so, transcription elongation complexes (TECs) were stalled within the leader region such that some or all of the 11(G/U)AG repeats of the TRAP binding site were exposed on the nascent transcript. TRAP was then allowed to bind to the exposed RNA from the TEC, after which the ability of AT to prevent TRAP-mediated transcription termination was assessed.

Our results indicate that AT can prevent TRAP-mediated termination at the *trp* attenuator after TRAP has bound the nascent RNA as long as it has not bound to all 11 (G/U)AG repeats. Under these circumstances, AT prevents TRAP-mediated termination by inducing TRAP to dissociate from the nascent *trp* mRNA. Our data also suggest that the 5′-most region of the TRAP binding site RNA is most susceptible to dissociation from TRAP. We also provide evidence that a complex of one AT trimer bound to a TRAP 11mer has significantly lower affinity for its target RNA than WT TRAP. Moreover, when examined using an *in vitro* transcription system, this AT-TRAP complex does not induce termination in the *trp* leader region.

In this report we have revealed an additional and/or alternate mechanism through which AT modulates TRAP-mediated attenuation control of the *trp* operon in *B. subtilis*. Our findings add to the existing body of knowledge of gene regulation in bacteria. In addition, our studies may have broad implications in understanding how critical processes in both single-celled and multicellular organisms may be governed through interacting oligomeric proteins.

## MATERIALS AND METHODS

### Materials

Plasmids used in these studies were propagated in *Escherichia coli* K802. Templates for *in vitro* transcription were based on the plasmid pUC*trpL*, which contains a 730-bp EcoRI-HindIII fragment (−411 to +318 relative to start of transcription) including the *B. subtilis trp* promoter, the regulatory leader region and the first 40 codons of the *trpE* gene (7,20). *B. subtilis* RNA polymerase was isolated from the strain MH5636 as described previously (21) with the exception that 30% trehalose was added to the storage buffer.

WT AT as well as AT containing a Cys substitution at residue 7 (D7C AT) were purified as described by Chen and Gollnick (19). His-tagged N20C (Asn20 replaced with Cys) TRAP was purified using Ni-agarose according to the manufacturer's guidelines (Qiagen).

**Figure 1.**
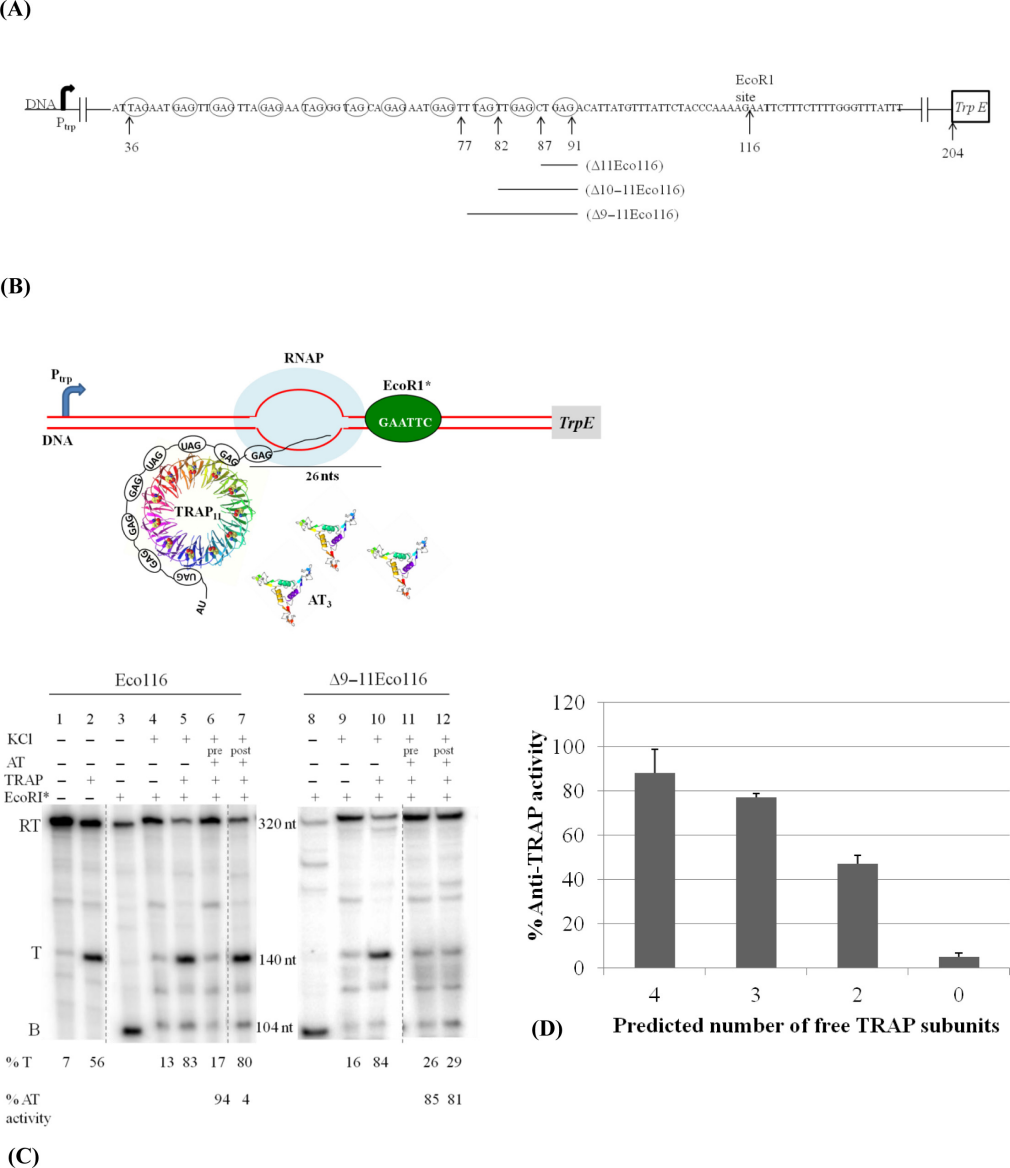
AT can modulate TRAP-mediated termination at the *trp* attenuator when TRAP is bound to fewer than 11 (G/U)AG repeats. (**A**) Diagram of the Eco116 template for *in vitro* transcription. This template encodes the entire *trp* leader region including the 11 (G/U)AG repeats of the TRAP binding site, each of which is circled, and the first 40 codons of the *trpE* gene (depicted as a box). The leader region is capable of forming two mutually exclusive RNA secondary structures, the antiterminator from +60 to +111, or an overlapping terminator (attenuator) from +108 to +133 (all numbers are relative to the start of transcription). An EcoRI site was created by site directed mutagenesis beginning at G116. Similar templates were created that contain deletions (represented as black lines below the nucleotide sequence) of one to three of the 3′-most triplet repeats of the TRAP binding site (Δ11Eco116, Δ10–11Eco116 and Δ9–11Eco116). (**B**) Schematic diagram of transcription elongation complex (TEC) blocked by EcoRI* on the Δ9–11Eco116 template in the presence of AT. The double-stranded DNA template is represented as two red lines and the *trp* promoter region is shown as a blue arrow. RNAP is depicted as a light blue oval and EcoRI* as a green oval bound to its recognition site on the DNA template. TRAP is depicted in ribbon diagrams with each subunit in a different color and the bound tryptophans represented as Van der Waals spheres. TRAP is shown bound to 7 (G/U)AG repeats exposed on the nascent transcript of the blocked TEC. Three AT trimers are depicted in ribbon diagrams with each subunit in a different color. (**C**) PAGE analysis of the products of *in vitro* transcription of the Eco116 (lanes 1–7) or Δ9–11Eco116 (lanes 8–12) templates in the absence or presence of TRAP (50 nM), AT (80 μM) and EcoRI* (130 nM) as indicted above the gel. Transcription of these templates yields three major transcripts corresponding to TECs that are blocked by EcoRI* (B), terminated within the leader region (T) or that read through to the end of the template (RT). Pre and post refers to incubating AT with TRAP prior to addition to the transcription reaction (pre) or adding AT after TRAP has been allowed to bind to the nascent transcript (post). The percent transcription termination (% T) is shown below each lane. This experiment was repeated three times with standard deviations of less than 6%. The percent AT activity is indicated at the bottom of each lane below the % T. AT activity is defined as the ability of AT to prevent TRAP-mediated termination (see the Materials and Methods section). All lanes are a part of the same gel but other lanes have been cropped out, and the dashed lines depict the cropped region. (**D**) Bar graph of the percent AT activity when AT is added after TRAP has bound the nascent transcript on the blocked TEC. The number of free subunits is based on the assumption that TRAP has bound to all of the exposed triplet repeats on the exposed RNA. Values shown are the average of three independent trials. Standard deviations are indicated for each bar.

The E111Q mutant EcoRI (EcoRI*) protein was purified as described by Potter *et al.* (7). This mutant protein retains the ability to bind to DNA but is deficient in cleavage activity (22). EcoRI* bound to DNA blocks the TEC such that the nascent RNA is exposed from RNA polymerase up to 26–27 bases upstream of the G of its GAATTC recognition site (23–25). We used EcoRI* to block the TEC during *in vitro* transcription of the *trp* leader region on several DNA templates. The first of these templates, termed *trpL*Eco116, was constructed from the plasmid pUC*trpL* by creating an EcoRI recognition site (GAATTC) starting at position +116 relative to the start of transcription (Figure 1A). This EcoRI site was introduced by making a G to T substitution at residue +119 (G119T) in the leader sequence using the QuikChange Site-Directed Mutagenesis kit (Stratagene) to create pUC*trpL*Eco116. EcoRI* bound to this template blocks the TEC such that the nascent *trp* transcript is exposed from +1 to ∼ +90, which includes nearly the entire TRAP binding site including the first 10 triplets and the first two residues (GA) of the 11th repeat.

Templates from which subsets of the 11 triplet repeats of the TRAP binding site are exposed on the nascent transcript when the TEC is blocked by EcoRI* were created based on pUC*trpL*Eco116. Attempts to create these templates by placing the EcoRI recognition site upstream of +116 were unsuccessful because EcoRI* blocking the TEC at these sites was inefficient. The reason for this inefficient blocking is not known but it may involve the influence of the DNA sequences surrounding the GAATTC site on the affinity of EcoRI* binding (26). Hence we used an alternative approach of deleting segments of the *trp* leader region at the 3′-end of the TRAP binding site between +76 and +92 (relative to the start of transcription) in the Eco116 template (Figure 1A). These templates were created by using QuikChange mutagenesis (Stratagene) to delete DNA segments of the Eco116 template that encode the 11th (+87 to +91), 10th and 11th (+82 to +91) or 9th–11th (+77 to +91) repeats. These deletions generated the plasmids pUC*trpL*Δ11Eco116, pUC*trpL*Δ10–11Eco116 and pUC*trpL*Δ9–11Eco116 respectively, which were subsequently used for making templates for *in vitro* transcription (see below). When the TEC is blocked by EcoRI* on these templates, 9, 8 or 7 of the (G/U)AG repeats of the TRAP binding site are exposed on the nascent transcript respectively.

Templates for *in vitro* transcription were created by polymerase chain reaction (PCR) using the plasmids described above. In each case, the primers used were the standard M13 Reverse primer and a primer corresponding to −104 to −83 of the *trp* leader region (relative to the start of transcription) (Integrated DNA Technologies). The PCR products were purified by phenol-chloroform extraction followed by ethanol precipitation. When transcription templates were created to bind to streptavidin-coated beads, the 5′-end of the M13 Reverse primer was biotinylated (Integrated DNA Technologies). The PCR products were purified as described above and the resulting 5′ biotinylated templates were coupled to streptavidin-coated paramagnetic beads (Promega) according to the manufacturer's instructions.

### Filter binding assay for RNA binding to TRAP

A previously developed filter binding assay was used to measure RNA binding to TRAP or to AT-TRAP complexes (27). The 55 nt RNA used in these assays consisted of the sequence GAGUU repeated 11 times. This RNA was generated by T7 polymerase transcription of HindIII linearized pTZ18GAGUU in the presence of α-^32^P UTP (27). Increasing amounts of TRAP or TRAP-AT were mixed with 1 fmol of ^32^P-labeled (GAGUU)_11_ RNA (∼5000 dpm) and 10 μg yeast RNA in filter binding buffer (16 mM Tris pH 8.0, 250 mM KCl) containing 1 mM tryptophan. After incubation for 20 min at 37°C, 40 μl aliquots from each 50 μl reaction were filtered through a two membrane layer using a 96-well dot blot mini fold apparatus (Schleicher and Schuell). The membranes consisted of 0.45 μm nitrocellulose (Schleicher and Schuell), which retains protein and any RNA bound to the protein, on top of Hybond-N+ (Amersham), which retains unbound RNA. Each reaction was washed with 80 μl filter binding buffer. The membranes were dried and exposed to a phosphorstorage screen (GE Healthcare), which was scanned with a Storm Phosphorimager (GE Healthcare). Quantification of each assay was performed using ImageQuant Software (GE Healthcare). For each reaction, the amount of RNA on the nitrocellulose divided by total RNA on nitrocellulose and Hybond was used to calculate the fraction of RNA bound to TRAP. The data were analyzed using a non-linear single binding-site regression algorithm (Prism, GraphPad Software Inc.) to determine apparent dissociation constant (*K*_D_) values.

### *In vitro* transcription attenuation assay

The effect of AT on TRAP-mediated termination at the *trp* attenuator was examined using an *in vitro* transcription attenuation assay based on the method described by Potter *et al.* (7). For reactions that involved blocking TECs, EcoRI* (130 nM) was incubated with the DNA template (20 nM) in transcription buffer (20 mM Tris-HCl pH 8.0, 6 mM MgCl_2_, 2 mM DTT, and 100 mM KCl) for 5 min at 37ºC prior to initiating transcription. Transcription was initiated by addition of RNA polymerase (RNAP) (50 μg/ml) together with 8 μM ATP and GTP as well as 2 μM UTP and 1 μCi [α-^32^P] UTP (3000 Ci/mmol), followed by incubation for 10 min at 37°C. Heparin (0.1 mg/ml) was then added to prevent re-initiation, and transcription was continued for 10 min at 37°C in the presence of 16 μM NTPs and 1 mM L-tryptophan either in the absence of TRAP, the presence of TRAP, or with TRAP that had been pre-incubated with AT.

To examine the ability of AT to interact with TRAP before it has bound to the nascent *trp* transcript, AT and tryptophan-activated TRAP were combined prior to addition to the transcription reaction. To examine AT function after TRAP had bound the nascent *trp* transcript, AT was added after TRAP had been incubated in these reactions for 10 min at 37°C. For transcription reactions involving dissociation of EcoRI* from the DNA, 0.5 M KCl was added followed by an additional 10 min incubation at 37°C. All reactions were stopped by addition of an equal volume of stop solution (95% formamide/20 mM EDTA, pH 8.0/0.3 mg/ml each of bromophenol blue and xylene cyanol). Samples were denatured at 95°C for 2 min and run on 6% polyacrylamide-8 M urea gels at 20 mA for 1 h. Gels were dried and exposed to a phosphorstorage screen, scanned and quantified as described in the previous section.

To allow comparisons of the effects of AT on TRAP when different DNA templates were used, we defined AT activity as the ability of AT to prevent TRAP-mediated termination. To assess AT activity, we first measured TRAP-mediated termination for each template as the increase in the percentage of TECs that terminate in leader region in the presence of TRAP above that observed in the absence of TRAP. AT activity was then calculated as the ratio of the decrease in TRAP-mediated termination in the presence of AT compared to TRAP-mediated termination in the absence of AT.

### TRAP pull down assay

Pull down assays were performed to examine association of TRAP with the nascent RNA in the absence or presence of AT. Biotinylated DNA templates for *in vitro* transcription were constructed that expose either all 11 or subsets of the (G/U)AG repeats in the *trp* leader region as described above. *In vitro* transcription reactions were performed using these bead-bound templates as described above but in the absence of radiolabeled UTP, and the reactions were scaled up 8-fold. After transcription was allowed to elongate to the EcoRI* roadblock, TRAP was added followed by a 10 min incubation at 37°C. AT was added to TRAP either before or after it was allowed to bind to the nascent transcript, followed by a 10 min incubation. Bead-bound TECs were separated from the solution with a magnet and washed twice with transcription buffer containing 1 mM L-tryptophan to remove unbound TRAP. After washing, the beads were resuspended in transcription buffer with 0.1 mg/ml DNaseI and incubated for 15 min at 37°C to free the ternary complexes from the bead. These supernatants were filtered through a nitrocellulose membrane using a Minifold system (Schleicher and Schuell).

The amount of TRAP associated with the ternary complexes was determined by immunoblotting using polyclonal rabbit antibodies against *B. subtilis* TRAP followed by goat anti-rabbit IgG secondary antibodies conjugated with horseradish peroxidase (Bio-Rad). Detection was with Amersham ECL Plus using a Storm 860 (GE Healthcare) at 450 nm. Images were quantified using ImageQuant software (GE Healthcare). For each template, the amount of background TRAP pulled down in the absence of EcoRI* was subtracted from the amount measured with the blocked TECs on each template. The amount of TRAP pulled down when the TEC was blocked with EcoRI* in the absence of AT was set to 100%.

Similar pull down assays were performed to examine the susceptibility of the 5′ or 3′ region of the RNA binding site to dissociate from TRAP. For these assays, instead of adding AT, DNA oligonucleotides were used to compete with TRAP for binding to the nascent RNA. 200 nM DNA oligonucleotides (oligo) were added to the transcription reaction after allowing TRAP to bind to the nascent transcript followed by 10 min incubation at 37°C. The following oligos were used: 5′ antisense, complementary to either 3 (G/U)AG triplet repeats of the 5′-most portion of the TRAP binding site (+32 to +52 relative to the start of transcription); 3′ antisense, complementary to 3 (G/U)AG repeats of the 3′-most portion of the TRAP binding site (+66 to +82 or +61); 5′ sense, containing the same sequence as the first three (G/U)AG repeats of the binding site (+32 to +52), and a non-specific oligo, containing 102 nt of random sequence with no similarity to the TRAP binding site.

### Cross-linking and purification of cysteine substituted TRAP and AT proteins

Bismaleimidoethane (BMOE) was used to crosslink AT and TRAP. BMOE is a homobifunctional crosslinker for covalent, irreversible crosslinking between sulfhydryl groups (Pierce). The distance between the functional groups in BMOE is 8 Å and therefore only cysteine (Cys) residues in close proximity are crosslinked. In the crystal structure of the AT-TRAP complex the side chains of Asn 20 of TRAP and Asp 7 of AT are at an appropriate distance and orientation to be crosslinked by BMOE (18). We introduced cysteine substitutions at position 20 of TRAP and at position 7 of AT using the QuikChange kit (Stratagene) to generate N20C TRAP and D7C AT respectively.

Prior to the crosslinking reaction, N20C TRAP and D7C AT were passed through a G-25 Sephadex spin column equilibrated with crosslinking buffer (PBS pH 7.2 and 0.5 mM EDTA). N20C TRAP (0.02 mM) and D7C AT (0.08 mM) were mixed in the presence of 1 mM tryptophan in crosslinking buffer and BMOE (0.04 mM) followed by incubation at 37°C for 1 h. The reactions were quenched with 2 mM DTT at 37°C for 15 min. Crosslinking was analyzed on 15% SDS gels run at 60 mA for 1 h and then stained with Coomassie blue.

To generate specific complexes with one AT trimer bound to each TRAP 11mer, we created TRAP hetero-11mers composed of one N20C subunit and 10 WT subunits (20,28). To do so, a limiting amount of N20C TRAP in which each subunit contains six histidine residues at the carboxy terminus was mixed with 50-fold excess of WT TRAP. The proteins were denatured with 5 M guanidine-HCl for 1 h at room temperature and then renatured by dialysis in 50 mM phosphate buffer pH 8.0 overnight. We have previously shown that this procedure allows WT and mutant subunits to assemble into 11mers randomly. Due to the large excess of WT subunits used, virtually none of these hetero-11mers contain more than one His-tagged N20C subunit (28). Hetero-11mers containing one His-tagged N20C subunit and 10 WT subunits, (N20C)_1_(WT)_10_, were purified on Ni agarose. Since only the N20C subunit can crosslink with D7C AT, only one AT trimer can crosslink to each (N20C)_1_(WT)_10_ TRAP hetero-11mer. These TRAP hetero-11mers were mixed with 5-fold excess D7C AT in crosslinking buffer and crosslinked with BMOE as described above. Complexes containing D7C AT crosslinked to (N20C)_1_(WT)_10_ TRAP were purified on two successive immunoaffinity chromatography columns. The first contained antibodies against *B. subtilis* TRAP (6) and the second contained antibodies against *B. subtilis* AT. The TRAP affinity column removes unreacted D7C AT from the crosslinking reaction. Unreacted TRAP was then removed by the AT immunoaffinity column, yielding the crosslinked D7C AT- (N20C)_1_(WT)_10_ TRAP complex, which is referred to as TRAP-AT = 1:1.

## RESULTS

### AT can modulate TRAP-mediated termination when TRAP is bound to fewer than 11 (G/U)AG repeats

Prior studies of isolated TRAP and AT have shown that AT can prevent TRAP from binding to the *trp* leader RNA but cannot dissociate RNA from TRAP after it has bound (17). These observations suggest that AT can only regulate *trp* gene expression by interacting with free TRAP prior to binding to the leader region of the *trp* mRNAs. However, there are several features of these studies that may differ from the situation *in vivo*. In order to mediate transcription attenuation control of the *trp* operon, TRAP must bind to the nascent *trp* transcript (2). TRAP likely binds sequentially to the 11 (G/U)AG repeats as they become exposed from RNAP during transcription of the leader region. The goal of the studies presented here was to determine whether AT can modulate TRAP-mediated control of attenuation after TRAP has initiated binding to the 11 triplet repeats of the binding site on nascent RNA.

TRAP can stably bind to RNAs that contain 7 or more (G/U)AG triplet repeats (29, K. Potter, unpublished observation). We created several DNA templates for *in vitro* transcription that contain the *trp* promoter, the regulatory leader region and the start of *trpE*, the first structural gene of the *trp* operon (Figure 1A). Each template contains an EcoRI restriction site such that when the TEC is stalled by EcoRI* bound to the DNA 7, 8, 9 or approximately 11 of the triplet repeats are exposed on the nascent transcript. When TRAP is bound to the nascent RNA from these blocked TECs, approximately 4, 3, 2 or 0 subunits, respectively, remain unbound by RNA and are thus available to potentially interact with AT (Figure 1B). We used this system to examine the ability of AT to interfere with transcription termination after TRAP was bound to various number of triplet repeats. To do so, TECs were blocked with EcoRI*, TRAP was added and allowed to bind to the nascent transcript, then AT was added, and finally KCl (0.5 M) was added to dissociate EcoRI* from the DNA template and allow transcription to resume. This concentration of KCl does not affect TRAP binding to RNA (27) and the TEC is allowed to resume elongation (24,25). The results of these transcription reactions were compared to reactions in which the TEC was blocked by EcoRI* and released in the absence of TRAP or when AT and TRAP were combined prior to their addition to the transcription reaction (Figure 1C).

Transcription of the Eco116 template produced two major RNAs corresponding to transcripts that terminate at the *trp* terminator/attenuator (Figure 1C, *T* ≈ 140 nt), and those that read through the attenuator and continue to the end of the template (Figure 1C, RT ≈ 320 nt). In the absence of TRAP, transcription yielded virtually completely (93%) read-through transcripts (Figure 1C, lane 1). In the presence of tryptophan-activated TRAP, the fraction of transcripts terminating at the attenuator increased to 56% (Figure 1C, lane 2), indicative of TRAP-mediated termination at the attenuator. These results are similar to those seen with WT *trpL* template (7,8,20), indicating that the presence of the EcoRI recognition site did not significantly alter the regulatory properties of the leader region.

In the presence of EcoRI*, greater than 85% of the TECs were blocked, yielding an approximately 104 nt transcript (Figure 1C, lane 3, *B* ≈ 104 nt). Since 14 nt of the nascent transcript are within the blocked TEC (24), ∼ 90 nt are exposed on the nascent RNA, which includes the first 10 (G/U)AG repeats as well as the first two residues (GA) of the 11th repeat (Figure 1A). For convenience, we will describe this complex as having 11 repeats exposed on the nascent RNA. When EcoRI* was dissociated from the DNA template with 0.5 M KCl, and transcription was allowed to resume in the absence of TRAP, the majority of the TECs continued past the attenuator yielding only 13% terminated transcripts (Figure 1C, lane 4). When transcription was resumed after TRAP was allowed to bind to the nascent transcript on the blocked TEC, the majority (83%) of the transcripts terminated at the attenuator (Figure 1C, lane 5). AT inhibited the ability of TRAP to induce termination when the two proteins were incubated in the presence of tryptophan prior to addition to the stalled TEC (Figure 1C, lane 6). However, AT did not significantly affect TRAP-mediated termination when it was added to the transcription reaction after TRAP was allowed to bind to the nascent transcript, in which case 80% of the transcripts terminated at the attenuator (Figure 1C, lane 7). These observations are all consistent with prior studies (17,19), which showed that AT could bind to TRAP and prevent it from binding to RNA but AT could not dissociate the RNA-TRAP complex.

Transcription of the Δ9–11Eco116 template in the presence of EcoRI* resulted in ≈97% of the TECs being blocked (Figure 1C, lane 8), which yields an 83 nt transcript. Approximately 69 nt are exposed on this nascent RNA including 7 (G/U)AG repeats. Similar to the results with the Eco116 template, when EcoRI* was dissociated from the Δ9–11Eco116 template and transcription was resumed in the absence or presence of TRAP, 16% or 84% of the transcripts terminated at the attenuator respectively (Figure 1C, lanes 9 and 10). Again, when AT was added to TRAP prior to its binding to the nascent *trp* transcript, it prevented TRAP-mediated termination (Figure 1C, lane 11). However, in contrast to the results with the Eco116 template (Figure 1C, lane 7), in this case when AT was added after TRAP was bound to the nascent transcript, and then transcription was resumed, only ∼29% of the transcripts terminated at the attenuator, while the remainder continued to the end of the template (Figure 1C, lane 12). Hence with this template, AT was able to interfere with TRAP-mediated transcription termination after TRAP had bound to the ∼7 exposed (G/U)AG repeats on the nascent transcript. In this situation, ∼4 TRAP subunits are not bound to the RNA (Figure 1B).

Similar *in vitro* transcription attenuation assays were performed with templates on which 8 or 9 (G/U)AG repeats of the TRAP binding site are exposed on the nascent transcript from the stalled TEC. When TRAP binds to these transcripts, approximately 3 or 2 subunits of the TRAP 11mer should be unbound respectively. When AT was added after TRAP had bound to the nascent transcript, the AT activity correlates with the number of free TRAP subunits that are not predicted to be bound by RNA (Figure 1D). AT activity was greatest (80–90%) when 3–4 TRAP subunits are predicted to be free of RNA in the TRAP-TEC complex (Figure 1D). AT activity dropped to approximately 50% when 2 TRAP subunits are predicted to be unbound by RNA (Figure 1D), and when all 11 TRAP subunits are bound to the RNA and thus 0 subunits are free to bind AT in the complex (Figure 1D), AT activity was less than 10%. Hence it appears that in order for AT to influence TRAP-mediated termination after TRAP has initiated binding to the nascent transcript, it must do so before TRAP has bound to all 11 (G/U)AG repeats.

### AT causes TRAP to dissociate from RNA when TRAP is bound to fewer than 11 (G/U)AG repeats

**Figure 2.**
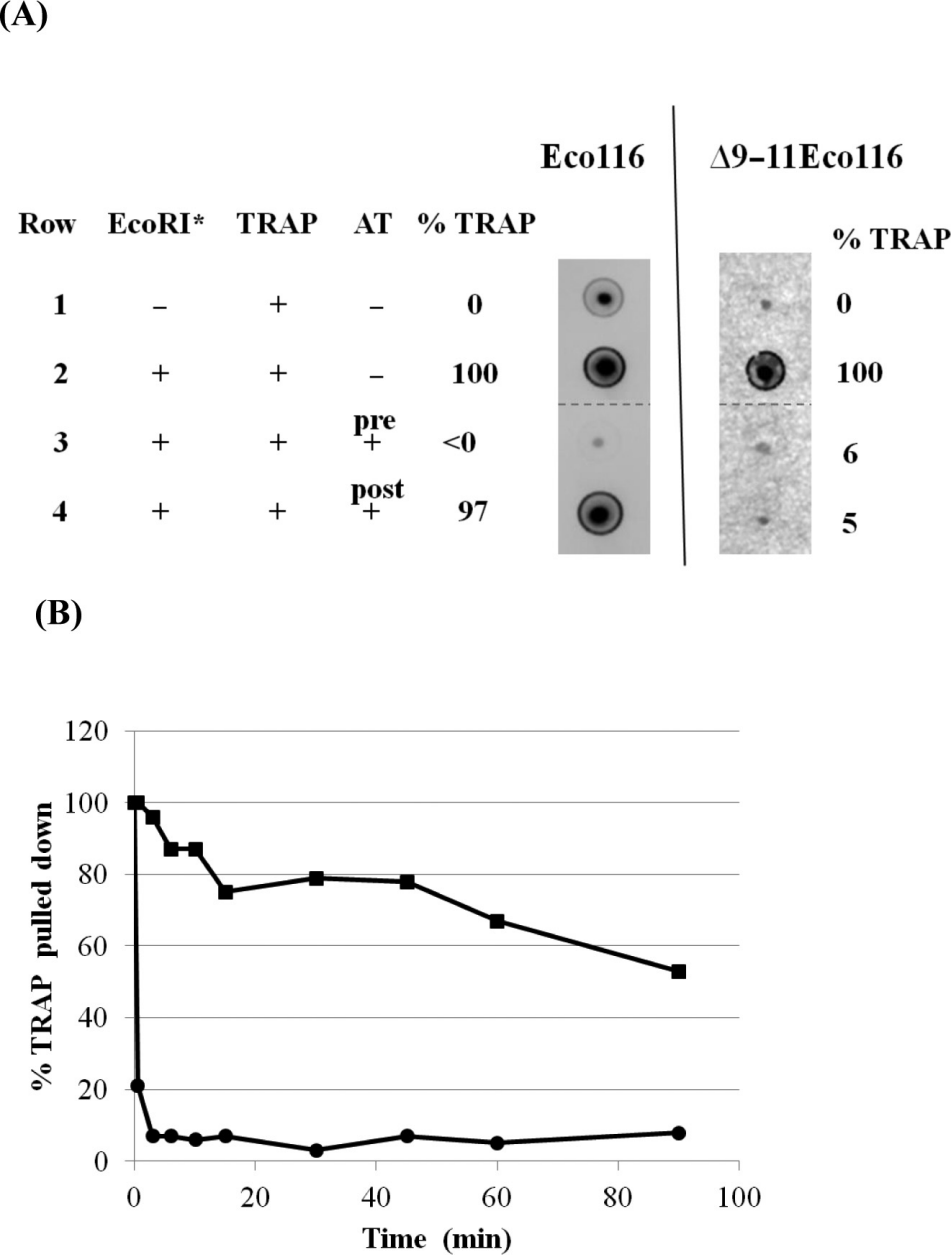
AT can induce dissociation of TRAP from the nascent *trpL* RNA. (**A**) Analysis of the amount of TRAP bound to the nascent transcript from blocked TECs in the absence or presence of AT. TRAP was added to blocked TECs on bead-bound templates, either Eco116 in left panel or Δ9–11Eco116 in right panel, in the absence or presence of AT. The templates were pulled down and the amount of TRAP remaining associated with the nascent RNA was measured by binding to nitrocellulose membranes followed by immunoblotting (see the Materials and Methods section). Pre refers to AT pre-incubated with TRAP for 10 min prior to addition to the transcription reaction, and post refers to AT added after TRAP was bound to the nascent RNA. [TRAP] = 20 nM, [AT] = 32 μM. The amount of TRAP pulled down in the absence of the EcoRI* (Row 1) was set as background. The amount of TRAP pulled down in the presence of EcoRI* in the absence of AT (Row 2) was set at 100% for each template. All rows are a part of the same experiment but other regions of the blot were removed from the figure. The borders of the removed portions of the blot are shown as dashed lines. (**B**) Graph of the percentage of TRAP associated with the nascent RNA from the Δ9–11Eco116 template, which contains 7 (G/U)AG repeats in the absence (squares) or in the presence (circles) of AT as a function of time (min). The amount of TRAP at time 0 was set as 100%.

There are several potential mechanisms by which AT could prevent TRAP-mediated termination at the *trp* attenuator after TRAP has initiated binding to the nascent RNA. In general, AT could prevent termination while TRAP remains bound to the RNA, or AT could induce TRAP to dissociate from the nascent RNA. To distinguish between these possibilities, we performed pull down assays to assess whether TRAP remains bound to the nascent transcript after AT is added to the TEC-TRAP complex. The templates Eco116 and Δ9–11Eco116 were used for these studies. These two templates were chosen because when added after TRAP was bound to the nascent transcript, AT showed significant ability to affect TRAP-mediated termination with the Δ9–11Eco116 template but not with the Eco116 template (Figure 1C). In each case, after the TECs were blocked by EcoRI*, TRAP and/or AT were added and the amount of TRAP that remained associated with the bead-bound TEC was assessed by immunoblotting. With both templates the amount of TRAP bound in the absence of AT when the TEC was blocked by EcoRI* was set to 100% (Figure 2A, Row 2). As expected, pre-incubation with AT prevented TRAP from binding to the nascent transcript from TECs stalled on either template (Figure 2A, Row 3). Addition of AT after TRAP was bound to the nascent transcript had no significant effect on the amount of TRAP associated with the TEC on the Eco116 template but resulted in dissociation of virtually all TRAP from the TEC on Δ9–11Eco116 (Figure 2A, Row 4).

The observed dissociation of TRAP from the nascent transcript of the Δ9–11Eco116 template in the presence of AT could be the result of AT directly mediating dissociation of the TRAP-TEC complex, or it could be due to AT binding to TRAP after it dissociates from the nascent transcript and preventing it from rebinding to the RNA. To distinguish between these possibilities, we examined the time course of TRAP dissociating from the nascent RNA on stalled TECs on the Δ9–11Eco116 template in the absence or presence of AT (Figure 2B). TRAP was allowed to bind to the nascent RNA on the blocked TEC for 10 min, after which the bead-bound TECs were washed twice and resuspended in transcription buffer containing tryptophan in the absence or presence of AT. Aliquots were removed at various time points and the amount of TRAP associated with the bead-bound TEC was determined by immunoblotting as described above.

In the absence of AT, TRAP displays a two-phase dissociation profile (Figure 2B). Approximately 20% of the bound TRAP dissociated during the first 20 min of incubation, followed by slower dissociation over the next 70 min. After 90 min, approximately 50% of the TRAP dissociated from the TEC. In the presence of AT, 80% of the initially bound TRAP dissociated from the TEC within 0.5 min and virtually all dissociated within 3 min (Figure 2B). These observations suggest that AT plays an active role in inducing TRAP to dissociate from the TEC.

These studies indicating that AT can induce dissociation of TRAP from the nascent RNA are consistent with the effects that AT had on TRAP-mediated transcription termination. In both cases, AT can influence TRAP function after it has bound to the nascent transcript on the Δ9–11Eco116 template but not with the Eco116 template (Figure 1C). The major difference between these two templates is the number of (G/U)AG repeats of the TRAP binding site that are exposed on the nascent RNA when the TEC is blocked by EcoRI*. Nearly the entire 11 repeat binding site is exposed on the transcript from the Eco116 template whereas only 7 (G/U)AG repeats are exposed on the transcript from Δ9–11Eco116. Hence when TRAP is bound to the nascent transcripts from Eco116 or Δ9–11Eco116, approximately 0 or 4 of its 11 subunits are predicted to remain unbound by RNA, respectively. These results suggest that the presence of several free TRAP subunits is necessary for AT to cause TRAP to dissociate from the nascent RNA during transcription of the leader region.

### The 5′-most region of the TRAP binding site RNA is most susceptible to dissociation from TRAP

**Figure 3.**
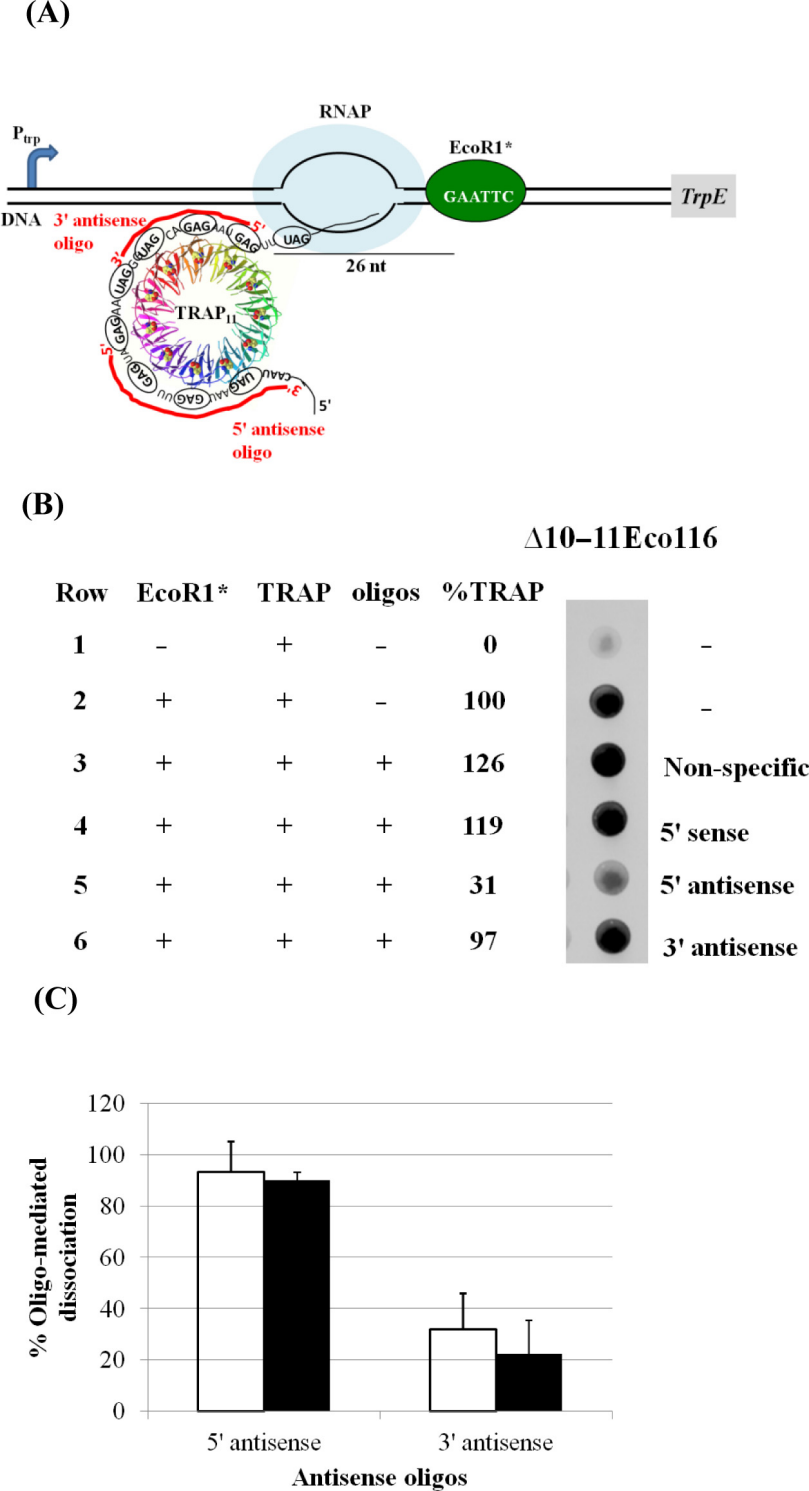
Effect of DNA oligonucleotides on the association of TRAP with the nascent *trp* leader RNA. (**A**) Schematic representation of the TEC blocked by EcoRI* on the Δ10–11Eco116 template. RNAP is depicted as a light blue oval and EcoRI* is shown as a green oval bound to its recognition site on the DNA template, which is indicated as two black lines. TRAP is depicted in ribbon diagrams with each subunit in a different color and the bound tryptophans as Van der Waals spheres. TRAP is bound to 8 (G/U)AG repeats exposed on the nascent transcript. DNA oligos complementary to (G/U)AG repeats of the 5′-most portion or the 3′-most portion of the TRAP binding site (5′ antisense and 3′ antisense, respectively) (described in the Materials and Methods section) are depicted as red lines. (**B**) Analysis of the amount of TRAP bound to the nascent RNA on blocked TECs in the absence of oligo or when an oligo was added after TRAP had bound to the nascent RNA. TECs on bead-bound Δ10–11Eco116 were pulled down and applied to a nitrocellulose membrane followed by immunoblotting with antibodies against *B. subtilis* TRAP (see the Materials and Methods section). [TRAP] = 20 nM, [oligo] = 200 nM. The background amount of TRAP pulled down in the absence of EcoRI* (Row 1) was subtracted from all other assays. The amount of TRAP pulled down in the presence of EcoRI* in the absence of oligo was set as 100% (Row 2). (**C**) Bar graph of the percentage TRAP dissociated from the nascent transcripts of the Δ10–11Eco116 (open bars) or Δ10–11Eco116EX (closed bars) templates in the presence of antisense oligos complementary to the 5′-most portion or 3′-most portion of the TRAP binding site (5′ antisense and 3′ antisense respectively). The Δ10–11Eco116EX template is similar to the Δ10–11Eco116 template except that it contains 16 residues of random sequence between the last repeat and EcoRI site. Values shown are the average of three independent trials with standard deviations indicated.

To further characterize the mechanism by which AT induces TRAP to dissociate from the nascent transcript, we examined whether the 5′- or the 3′-most portion of the binding site in the nascent RNA is more susceptible to dissociation. It is not possible to direct AT to influence TRAP binding specifically at either region of the RNA binding site. Hence, we used antisense DNA oligonucleotides to compete with TRAP for binding to either the 5′- or 3′-most portion of the RNA binding site (Figure 3A).

These studies were performed using the Δ10–11Eco116 template, on which 8 (G/U)AG repeats are exposed on the nascent transcript when the TEC is blocked by EcoRI* (Figure 3A). This arrangement permitted design of specific non-overlapping antisense oligos complementary to triplet repeats in the 5′-most or 3′-most regions of the RNA in complex with TRAP (Figure 3A; described in the Methods section). For these assays, TECs were blocked with EcoRI*, TRAP was allowed to bind to the nascent RNA, and then the oligonucleotides were added and the reaction mixture was incubated for 10 min prior to collecting the bead-bound TECs from the solution. The amount of TRAP remaining associated with the TECs was measured by immunoblotting as described in the previous section. The amount of TRAP specifically bound (after subtracting non-specific background in the absence of EcoRI*; Figure 3B, Row 1) to the blocked TEC on this template in the absence of oligonucleotide was set to 100% (Figure 3B, Row 2).

Addition of either a non-specific oligo, or a sense oligo with the same sequence as the 5′-most (G/U)AG repeats of the TRAP binding site (5′ sense), did not affect the amount of TRAP associated with the TEC (Figure 3B, Rows 3 and 4, respectively). This observation is consistent with prior studies which showed that TRAP does not bind single-stranded DNA with GAG or TAG repeats (30). In contrast, addition of antisense oligos complementary to (G/U)AG repeats at the 5′-most region of the RNA significantly reduced the amount of TRAP associated with the TEC (Figure 3B, Row 5 and Figure 3C, open bar, 5′ antisense respectively). Antisense oligos to the triplet repeats at the 3′-end of the binding site did not affect association of TRAP with the TEC (Figure 3B, Row 6 and Figure 3C, open bar, 3′ antisense respectively).

The observed differences in the effects of oligos targeted to the 5′ or 3′ regions of the TRAP binding site could be due to the 3′ region of the RNA being less accessible as a result of close juxtaposition of TRAP and RNAP in the blocked TEC (Figure 3A). To test whether close proximity of TRAP to RNAP in the stalled TEC affects the ability of antisense oligos to cause dissociation of TRAP, we created a template analogous to Δ10–11Eco116 but with 16 additional residues following the TRAP binding site before the EcoRI site. When RNAP is blocked on the Δ10–11Eco116EX template, there are 16 residues of random sequence between the last GAG repeat and RNAP in the nascent transcript. Antisense oligos to (G/U)AG repeats in the 5′-most region of the binding site again induced dissociation of TRAP (Figure 3C, closed bars, 5′ antisense) as was observed with the Δ10–11Eco116 template (Figure 3C, open bars, 5′ antisense). Again, antisense oligos targeted to the 3′-most repeats of the TRAP binding site induced significantly less dissociation of TRAP from the TEC (Figure 3C, open and closed bar, 3′ antisense).

Together these results indicate that within the TRAP-RNA-TEC complex, the 5′-most portion of the RNA of the TRAP binding site is more susceptible to dissociation from TRAP. These observations suggest that this portion of the RNA may be less stably bound to TRAP. If so, then AT may also initiate dissociation of TRAP from the 5′-most portion of the bound RNA.

### One AT trimer bound per TRAP 11mer reduces the affinity for RNA

Prior studies have shown that multiple AT trimers (AT_3_) can bind to each TRAP 11mer (18,19,31). However, it is not known how many AT trimers are required to bind in order to interfere with TRAP binding to RNA. Several lines of evidence suggest that as few as one AT_3_ bound per TRAP 11mer may significantly affect TRAP's ability to bind RNA and regulate the *trp* genes *in vivo.* Under conditions of severe tryptophan starvation, the presence of an estimated AT_3_ to TRAP 11mer ratio of less that 1:1 increased expression of the *trp* operon 30-fold as compared to the same conditions in the absence of AT (32). Similarly, our *in vitro* transcription studies using blocked TECs show that when TRAP is bound to the nascent RNA such that as few as two of its subunits are unbound by RNA, AT can prevent TRAP-mediated termination when added after TRAP has bound to the RNA (Figure 1D). The crystal structure of AT in complex with TRAP shows that one AT_3_ binds to two adjacent TRAP subunits (18). Although our studies were performed with excess AT, our observations suggest that as few as one AT_3_ binding per TRAP 11mer may be sufficient to initiate dissociation of TRAP from the nascent transcript.

We therefore examined the effect of one AT_3_ bound per TRAP 11mer on the ability of TRAP to bind (GAGUU)_11_ RNA. To do so, we used chemical crosslinking to create a complex with one AT trimer specifically bound to each TRAP 11mer. BMOE is a homobifunctional crosslinker specific for sulfhydryl groups. With 8 Å between the reactive maleimide groups, BMOE only crosslinks proteins with cysteine (Cys) residues in close proximity. WT TRAP does not contain any Cys residues (33), and while each WT AT subunit contains four Cys residues, they are all tightly complexed with Zn^2+^ (14) and do not react with BMOE (data not shown). This situation allowed us to create specific Cys substituted TRAP and AT proteins that can be crosslinked with BMOE.

**Figure 4.**
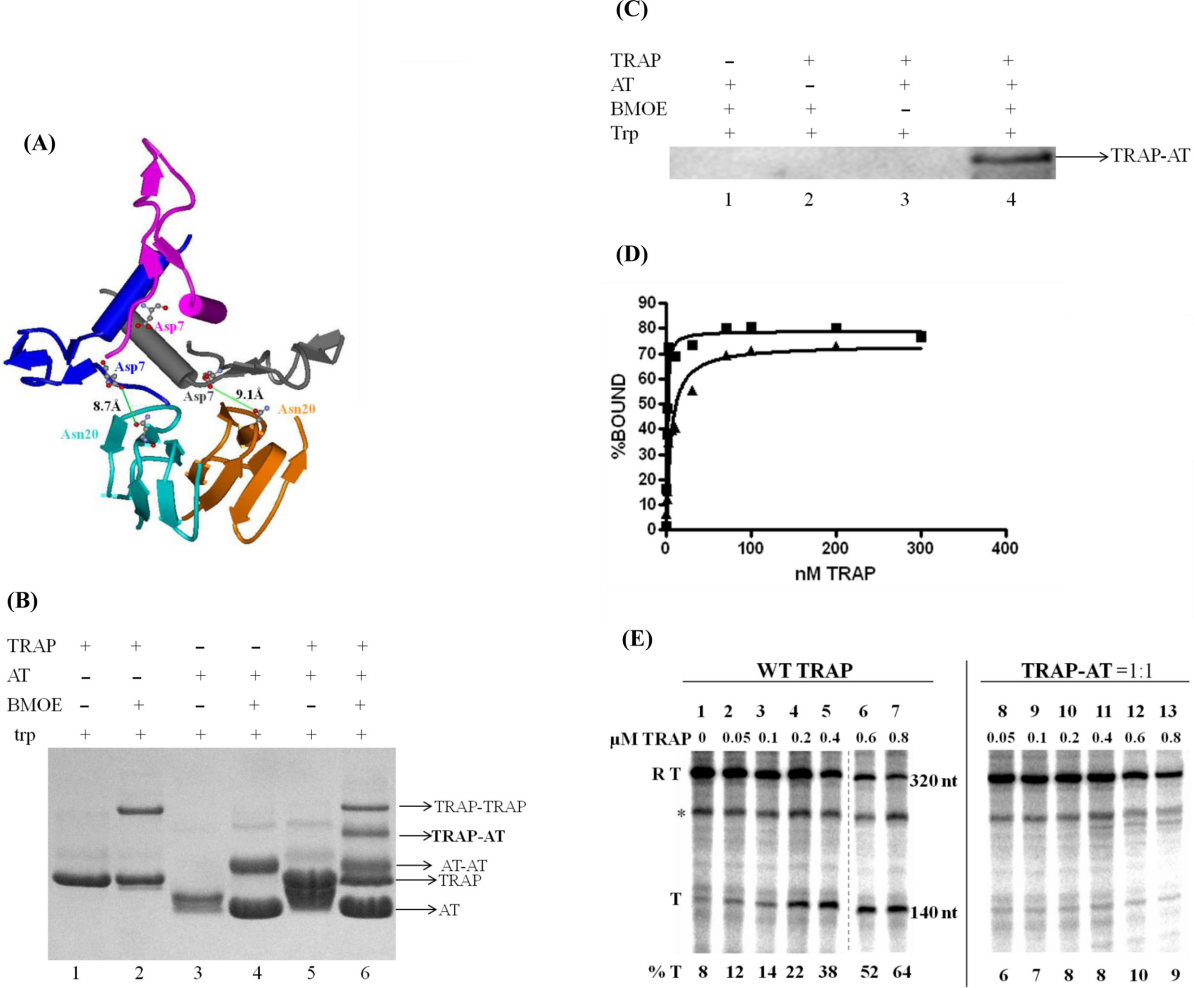
Analysis of the effect of one AT trimer bound to TRAP on RNA binding and TRAP-mediated termination *in vitro*. (**A**) Ribbon diagram depicting the AT-TRAP complex. Two TRAP subunits in blue and orange ribbon diagrams, and an AT trimer with blue, gray and pink ribbon diagrams for each subunit. This figure was generated with the coordinates from (18). Asn20 of TRAP and Asp7 of AT are depicted as ball and stick models. The distances between the side chains of Asn20 of TRAP and Asp7 of AT for two pairs of TRAP and AT subunits are shown. (**B**) SDS PAGE analysis of crosslinking between N20C TRAP and D7C AT by BMOE. Positions of TRAP monomers (TRAP), AT monomers (AT), self-crosslinked TRAP (TRAP-TRAP), self-crosslinked AT (AT-AT) and TRAP crosslinked to AT (TRAP-AT) are indicated. The samples were run on a 15% SDS gel and stained with coomassie blue. (**C**) Immunoprecipitation of the TRAP-AT complex. Samples containing N20C TRAP and/or D7C AT in the absence or presence of BMOE were precipitated with antibodies against *B. subtilis* TRAP followed by SDS PAGE and immunoblotting with polyclonal antibodies against *B. subtilis* AT. (**D**) Effect of one AT trimer bound to TRAP 11mer on the RNA binding affinity of TRAP. The curves represent TRAP (filled squares) or the TRAP-AT = 1:1 complex (filled triangles) binding to (GAGUU)_11_ RNA. The RNA binding curves shown are the best fit of the filter binding data and were analyzed using a non-linear single binding-site regression algorithm (Prism, GraphPad Software Inc.). The dissociation constant (*K*_D_) for WT TRAP binding to (GAGUU)_11_ RNA is estimated to be 0.55 ± 0.13 nM and that of TRAP:AT = 1:1 is 3.7 ± 1.6 nM. These values are an average of three independent trials. (**E**) PAGE analysis of the products of *in vitro* transcription of the WT *trpL* template in the absence or presence of increasing amounts of WT TRAP (lanes 1–7) or the TRAP-AT = 1:1 complex (described in the Methods section) (lanes 8–13). Positions of transcripts that read through the attenuator (RT = 320 nt) and transcripts that terminate at the *trp* attenuator (*T* = 140 nt) are indicated. A band running at approximately 250 nt of unknown origin is labeled with an asterisk (*). All lanes were part of the same gel but in the case of WT TRAP some lanes were removed from the figure and the dashed line depicts the border of the cropped region. The percentage of transcripts terminating at the *trp* attenuator (% T) is listed for each reaction and is indicated below each lane.

Based on examination of the crystal structure of AT in complex with TRAP (18), we identified two residues, Asn 20 in TRAP and Asp 7 in AT, with side chains at appropriate distances and juxtaposition in the complex to crosslink with BMOE. We then made Cys substitutions for each of these residues. Asn 20 of TRAP is located within the RNA binding region of TRAP but does not participate in binding RNA (Figure 4A (5)). Replacing Asn 20 with Cys (N20C) does not affect the ability of TRAP to bind RNA or AT (28). Asp 7 is located at the apex of the AT trimer (Figure 4A) and replacing Asp 7 with Cys (D7C) only slightly lowers the affinity of AT for TRAP (data not shown).

We first established that the N20C TRAP 11mer can be efficiently crosslinked with D7C AT using BMOE (Figure 4B). Interpretation of this gel is somewhat complicated because both N20C TRAP (Figure 4B, lane 2) and D7C AT (Figure 4B, lane 4) display self-crosslinking. Nevertheless, when a mixture of N20C TRAP and D7C AT is treated with BMOE, a band is seen that is not present when either protein alone is treated with BMOE (Figure 4B, lane 6: TRAP-AT). We confirmed that both proteins are present in this band by co-immunoprecipitation (Figure 4C). Moreover, when a mixture of N20C TRAP and D7C AT is treated with BMOE in the absence of tryptophan, this band is not seen (data not shown). Together these results show that N20C TRAP crosslinks with D7C AT (Figure 4B, lane 6 band: TRAP-AT).

To create a complex with one AT_3_ bound to each TRAP 11mer, we took advantage of our ability to create TRAP hetero-11mers composed of different types of subunits (28). Specifically, we created TRAP 11mers composed of 10 WT subunits and one N20C subunit (20). Since only the N20C subunit can crosslink with D7C AT, this arrangement allowed us to specifically crosslink one AT trimer to the single Cys-substituted subunit in the TRAP hetero-11mer. We purified the TRAP-AT complex from unreacted TRAP and AT using successive immunoaffinity columns with polyclonal antibodies raised against each protein. Only the AT-TRAP complex binds to both columns. Analysis of RNA binding of the TRAP-AT 1:1 complex to an RNA composed of 11 GAGUU repeats showed approximately 7-fold lower affinity (*K*_D_ = 3.7 nM) as compared to WT TRAP (*K*_D_ = 0.55 nM) (Figure 4D).

We further examined the effects of one AT_3_ bound per TRAP 11mer on its ability to induce transcription termination at the *trp* attenuator *in vitro*. Adding increasing amounts of tryptophan-activated WT TRAP increased the number of terminated transcripts accompanied by a decrease in the fraction of read-through transcripts, indicative of TRAP-induced termination at the *trp* attenuator (Figure 4E, lanes 2–7). In contrast, addition of up to 800 nM of TRAP-AT 1:1 complex did not significantly increase termination at the attenuator (Figure 4E, lanes 8–13). These results suggest that the presence of only one AT_3_ bound to a TRAP 11mer is capable of interfering with TRAP-mediated termination at the *trp* attenuator. On this gel a band running at approximately 250 nt is seen in all lanes (Figure 4E, marked *). This product was not reproducibly seen in our *in vitro* transcription reaction (see Figure 1C) and its identity is not clear. However, its production was not affected by the presence of TRAP (Figure 4E, lanes 1–7) or by the TRAP-AT complex (Figure 4E, lanes 8–13).

## DISCUSSION

AT binds to tryptophan-activated TRAP and prevents it from binding to its RNA targets, thereby increasing expression of the *trp* genes (10,17). Studies of isolated AT, TRAP and RNA showed that AT could not disrupt a preformed TRAP-RNA complex in which the RNA contained between 6 and 11 (G/U)AG repeats ((17), Y. Chen and P. Gollnick, unpublished data). These observations suggested that AT can only interact with the pool of free TRAP in the cell. However, *in vivo* TRAP interacts with the nascent transcript during transcription of the leader region to modulate transcription of the *trp* operon (2). We have used blocked TECs to represent intermediates during transcription of the leader region to show that AT can inhibit TRAP-mediated transcription termination after TRAP has initiated binding to its RNA target in the *trp* leader region but before it has bound to all 11 triplet repeats (Figure 1D). Moreover, under these circumstances AT induces dissociation of TRAP from the nascent RNA (Figure 2A).

The explanation for the differences in the observed ability of AT to induce TRAP to dissociate from isolated RNA as compared to a nascent transcript is not yet clear. However, it seems likely that the difference relates to the absence or presence of RNAP. Several observations suggest that TRAP interacts directly with RNAP to induce transcription termination. First, we have found that TRAP can induce efficient termination within a modified *trp* leader region lacking the entire segment that encodes the presumptive intrinsic terminator (9). Secondly, crosslinking studies of TRAP bound to the nascent *trp* transcript indicate that TRAP is in close proximity of RNAP when the TEC is blocked on the Eco116 template (C. Szyjka and P. Gollnick, unpublished observations).

Interaction between TRAP and RNAP may alter the manner in which TRAP binds to RNA so as to allow AT to induce dissociation of TRAP. Comparing the activity of AT on TRAP bound to the nascent transcripts from blocked TECs on the Δ10–11Eco116 and Δ10–11Eco116EX templates supports this proposal. The RNAs from these TECs expose nearly identical numbers of (G/U)AG repeats but on the former template the 3′-most repeat is immediately adjacent to RNAP whereas the Δ10–11Eco116EX template encodes 16 additional nucleotides between the last repeat and the EcoRI site on the DNA. Hence, TRAP is likely in close juxtaposition with RNAP on the Δ10–11Eco116 template (Figure 3A) but may be more distant from the polymerase on the Δ10–11Eco116EX template. Using the assay described in Figure 2, AT was far less effective in inducing dissociation of TRAP from the TEC with additional RNA between the TRAP binding site and RNAP (Supplementary Figure S1).

A recently identified TRAP mutant (E60K) has also provided evidence for a role of an interaction between TRAP and RNAP in influencing AT's ability to modulate TRAP function. E60K TRAP binds RNA and tryptophan with similar properties as the WT protein but fails to induce termination within the *trp* attenuator region (N. Merlino and P. Gollnick, unpublished observations). Hence, this mutant protein may contain a substitution in the region of TRAP that interacts with RNAP to induce transcription termination. AT is approximately 3-fold less efficient at inducing dissociation of E60K than WT TRAP from the nascent transcript from TECs blocked on Δ9–11Eco116 template (data not shown). Together these observations are all consistent with the suggestion that TRAP interacts directly with RNAP and that this interaction is important for AT-mediated dissociation of TRAP from the nascent transcript.

AT is approximately twice as effective at inducing dissociation of TRAP from stalled TECs that expose 7 or 8 (G/U)AG repeats as when 9 repeats are exposed (Figure 1D). The structure of TRAP in complex with AT suggests a possible explanation for these observations. Each AT trimer interacts with a pair of adjacent TRAP subunits. Moreover, when multiple AT trimers bind to TRAP, each AT trimer contacts the adjacent trimer, suggesting that cooperativity may be involved in AT binding to TRAP (18). When the TEC is blocked by EcoRI* on the Δ11Eco116 template, nine (G/U)AG repeats are exposed on the nascent transcript. In this situation, one AT trimer can bind to the remaining two free subunits of a TRAP 11mer bound to this RNA. When the TEC is blocked on the Δ10–11Eco116 template, 8 (G/U)AG repeats are exposed and as such when TRAP is bound to this nascent transcript three subunits would be available to interact with AT. In this case, one AT trimer can bind to two adjacent TRAP subunits and a second trimer may cooperatively interact with the first AT trimer and the third free TRAP subunit. Under these circumstances, the binding of the second AT trimer may displace the nascent RNA from the next TRAP subunit, thereby contributing to the observed increase in AT activity (Figure 1D). This model suggests that AT plays an active role in inducing TRAP to dissociate from the nascent transcript, which is consistent with our measurements of the rates of dissociation of TRAP from the nascent transcript from the Δ9–11Eco116 template (Figure 2B).

Using TECs blocked on the Δ10–11Eco116 template and antisense oligonucleotides, we have provided evidence that the 5′-most region of the TRAP binding site RNA is more susceptible to dissociation from TRAP than the 3′-most portion of the binding site (Figure 3B and C). Prior studies have shown that when TRAP binds RNA, it forms an initial complex with one or more triplet repeats near the 5′-end of the binding site followed by wrapping the remainder of the binding site in a 5′ to 3′ direction around TRAP (20,34). This directional binding of TRAP to RNA has been shown to be important for TRAP-mediated attenuation control of the *trp* operon (34). The 5′ to 3′ directionality of TRAP binding to RNA suggests that after the TRAP–RNA complex has formed, if the 3′ region of the binding site were to dissociate, it could re-anneal efficiently in the 5′ to 3′ direction. In contrast, if the 5′ region of the binding site RNA dissociated from TRAP, it would have to re-anneal in the 3′ to 5′ direction, which may not occur or may do so inefficiently relative to release of TRAP.

Several studies have shown that multiple AT trimers can bind to the TRAP 11mer (18,31). However, none of these studies have addressed how many AT trimers must bind in order to prevent TRAP from binding to RNA. Examining the effects of AT on *trp* gene expression *in vivo* has shown that ratios of less than one AT trimer per TRAP 11mer increase expression of the *trp* genes approximately 30-fold as compared to similar conditions in strains lacking AT (32). If multiple AT trimers must bind to prevent TRAP from binding to RNA, then these observations could be explained by AT binding to TRAP cooperatively. When the stoichiometry of AT_3_ and TRAP 11mer is 1:1, multiple (up to five) AT trimers would bind to a subset of the available TRAP 11mers. Alternatively, it is possible that binding of as few as one AT trimer can interfere with the ability of a TRAP 11mer to bind RNA and/or cause transcription to terminate in the *trp* leader region. To test this hypothesis, we created a model complex with one AT trimer bound per TRAP 11mer using site-specific chemical crosslinking. This complex showed approximately 7-fold lower affinity for RNA containing 11 (GAGUU) repeats (Figure 4D) as well as prevented TRAP-mediated transcription termination *in vitro* (Figure 4E). It is not possible to test this complex *in vivo* in order to determine the effect that this reduction in affinity for RNA would have on regulation of the *trp* genes. Hence we considered a mutant TRAP with a similar reduced affinity for RNA. H51A TRAP (His51 is substituted with Ala) also binds RNA with ∼7-fold lower affinity than WT TRAP, and this mutant protein shows ∼14-fold reduced regulation of a *trpE‘-’lacZ* fusion *in vivo* as compared to WT TRAP (5). This comparison would suggest that the presence of one AT_3_ bound to a TRAP 11mer may similarly reduce the ability of TRAP to regulate the *trp* operon.

An alternate explanation for the observed increase in expression of the *trp* operon at low ratios of AT_3_ to TRAP 11mer may be that each AT_3_ binds to multiple TRAP 11mers. Each trimer of AT contains three identical binding sites for TRAP (15). The crystal structure of AT in complex with TRAP involves only one of these three potential binding sites while the other two are free (18). Analytical ultracentrifugation (AU) studies have shown that mixing AT and TRAP at a ratio of 1:4 results in formation of a complex containing 12 AT subunits and 22 TRAP subunits, as well as the presence of large protein heterocomplexes of unknown stoichiometry (31). More recent studies of complexes formed at various AT_3_ to TRAP 11mer ratios using AU as well as small Angle X-Ray Scattering have provided further evidence for multiple TRAP 11mers that are linked by AT trimers at low ratios of AT_3_ to TRAP 11mer (35).

In this report, we have provided evidence for an additional mechanism through which AT modulates TRAP-mediated attenuation control of the *trp* operon in *B. subtilis*. Interaction of AT with TRAP during transcription of the leader region may provide a second opportunity for AT to prevent TRAP-mediated termination of the *trp* operon. Modulation of TRAP-mediated termination by AT after TRAP has initiated binding to the regulatory leader region may increase the likelihood that transcription reads through the *trp* attenuator so that the *trp* genes are expressed. AT-mediated regulation of the *trp* operon during transcription of the leader region may provide more flexibility to fine-tune tryptophan synthesis.

Gene regulation is indispensable for maintaining normal cellular function and for versatility and adaptability of an organism to its environment. Abnormal gene regulation is linked to various diseases. Therefore, fine-tuning the level of gene expression is necessary to maintain cellular homeostasis. Akin to the fundamental processes in eukaryotic cells, tryptophan metabolism in bacteria is highly regulated via RNA-protein and protein–protein interaction. These molecular interactions allow the cell to adapt to the environment depending on the availability of the nutrient tryptophan. Our findings may be applicable in understanding how crucial processes are regulated through interacting oligomeric proteins in both lower and higher organisms, including humans.

## SUPPLEMENTARY DATA

Supplementary Data are available at NAR Online.

SUPPLEMENTARY DATA
