# Supplementary material for: Modulating TRAP-mediated transcription termination by AT during transcription of the leader region of the Bacillus subtilis trp operon
Source: Nucleic Acids Res. 2014 Mar 20;42(9):5543–55. doi: 10.1093/nar/gku211 (PMC4027176; doi:10.1093/nar/gku211)
Supplement: SUPPLEMENTARY DATA [file supp_42_9_5543__index.html]

Modulating TRAP-mediated transcription termination by AT during transcription of the leader region of the Bacillus subtilis trp operon — SUPPLEMENTARY DATA 

# Modulating TRAP-mediated transcription termination by AT during transcription of the leader region of the *Bacillus subtilis trp* operon

## SUPPLEMENTARY DATA

**Files in this Data Supplement:**

- SUPPLEMENTARY DATA
