# Supplementary material for: Modulating TRAP-mediated transcription termination by AT during transcription of the leader region of the Bacillus subtilis trp operon
Source: Nucleic Acids Res. 2014 Mar 20;42(9):5543–55. doi: 10.1093/nar/gku211 (PMC4027176; doi:10.1093/nar/gku211)

**SUPPORTING DATA**

**Fig. S1. Effect of AT on the association of TRAP with nascent *trpL* transcript when there is additional RNA between TRAP binding site and RNAP. (A)** Analysis of the amount of TRAP bound to the blocked TEC in the absence or presence of AT pulled down on bead-bound ∆10-11Eco116 template (upper panel) or ∆10-11Eco116EX (lower panel) as measured by binding to nitrocellulose membrane followed by immunoblotting (see methods). [TRAP] = 20 nM, [AT] = 32 µM. The amount of TRAP pulled down in the absence of the EcoRI* was set as background (Row 1 and 4) and subtracted from all the assays for each template. The amount of TRAP pulled down in the presence of EcoRI* in the absence of AT was set at 100% for each template (Row 2 and 5). The dashed lines (dark grey) between rows 1-2, 2-3, 4-5 and 5-6 divide the figure into different parts of the same experiment

**Fig. S1.**


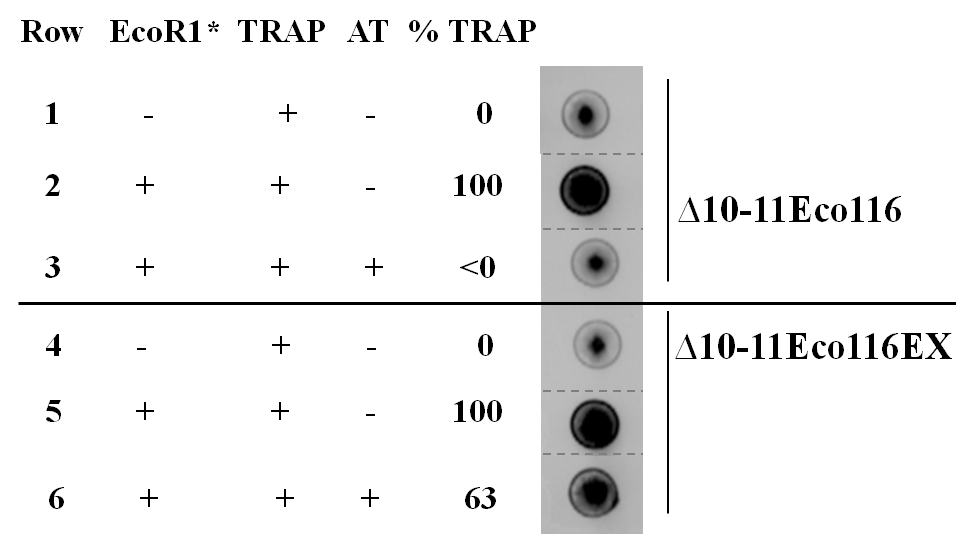

Supplement: SUPPLEMENTARY DATA [file supp_gku211_nar-00112-x-2014-File006.doc]
